# Pathological changes of testicular tissue in normal adult mice: A retrospective analysis

**DOI:** 10.3892/etm.2014.1481

**Published:** 2014-01-09

**Authors:** BAO-GUO XIE, JING LI, WEI-JIE ZHU

**Affiliations:** 1 Department of Developmental and Regenerative Biology, College of Life Science and Technology, Jinan University, Guangzhou, Guangdong 510632, P.R. China; 2 Department of Pathophysiology, Medical College, Jinan University, Guangzhou, Guangdong 510632, P.R. China

**Keywords:** mouse, testis, pathology

## Abstract

Mouse testicular experimental models are widely used in the study of andrology, reproductive toxicology and pharmacology. Under physiological conditions, a normal adult mouse is usually considered to have normal testes. However, whether normal adult mouse testes exhibit pathological changes has not been evaluated. The objective of this study was to investigate the pathological changes of testicular tissues in normal adult mice. A retrospective analysis of 720 adult male Kunming mice, used in previous studies as controls, was performed. Bilateral testicular tissues were stained with hematoxylin and eosin for pathological examinations. Among the 720 mice, nine had abnormal testes, an incidence of 1.3%. The nine mice with abnormal testes included two with microrchidia (22.2%) while the others had a normal testicular size. The observed pathological changes associated with microrchidia were seminiferous epithelial vacuolation, spermatogenesis arrest at the spermatocyte stage and the absence of sperm in all tubules. In other abnormal testes, pathological alterations included seminiferous epithelial vacuolation, severe hypospermatogenesis and symplasts composed of collapsed spermatids in tubules. The results demonstrate that normal adult male mice exhibit testicular pathological changes. Therefore, the possibility of abnormal testes in normal adult mice must be considered when using mice to establish a testicular experimental model.

## Introduction

Mouse testes have been established as a useful model for studies on andrology and reproductive toxicology (1,2). Under normal conditions, the mouse testis is comprised of a mixture of Sertoli and germ cells that work together to accomplish reproductive functions.

Spermatogenesis is a complex process of germ cell proliferation and differentiation. A large number of factors affect the process of spermatogenesis, including pathological changes of the seminiferous epithelium, aberrant gene expression and environmental factors (3). A previous study indicated that pathological changes of spermatogonia lead to impaired stretching of spermatids and damaged production of the axoneme in rats (4). Pathological changes of the seminiferous epithelium may cause the disruption of Sertoli and germ cells, which results in impaired spermatogenesis (5). Disruption of Sertoli cell function may also lead to germ cell loss (6). Moreover, an analysis of the seminiferous epithelium in mutant male mouse testis indicated that spermiogenesis may undergo arrest at various steps (7). Hence, pathological changes of the seminiferous epithelium may result in decreased spermatogenesis. However, whether normal adult male mouse testes exhibit pathological changes has not, to the best of our knowledge, been reported.

The aims of the present study were to investigate whether normal adult mouse testes exhibit pathological changes and to evaluate the incidence of testicular abnormalities in normal adult mice. A retrospective analysis of 720 adult male Kunming mice testicular tissues, used in previous studies as controls, was performed.

## Materials and methods

### Animals

A total of 720 healthy adult Kunming male mice (body weight, 29–36 g; age, 9–10 weeks) were purchased from the Medical Laboratory Animal Center (Guangzhou, China). These mice had all been used as normal controls in previous experiments between July 2006 and October 2011, and the testicular tissue samples taken in the previous experiments were analyzed in this retrospective study. Mice were maintained at a controlled temperature (23–25°C) and raised in a light-controlled room on a 12/12 h light-dark cycle. The mice were housed in metal cages and fed a standard laboratory diet. The protocol was approved by the Ethics Committee of Jinan University, Guangzhou, China

### Histological analysis

Mice were sacrificed by cervical dislocation and the testes were removed and placed in a Petri dish containing physiological saline. After washing, sections of the bilateral testicular tissues were quickly excised and then fixed in Bouin’s solution (Sigma, Louisville, KY, USA) for 24 h.

The samples were dehydrated and embedded in paraffin and 4-μm thick sections were cut and placed on glass slides, which were kept at 37°C for >12 h. The sections were immersed in xylol to remove the paraffin and then dehydrated with a descending alcohol series and deionized water. Finally, the sections were stained with hematoxylin and eosin prior to histological analysis.

## Results

In total, the testes of nine mice (1.3%) exhibited pathological changes in the study. Among the nine adult mice with abnormal testes, two of the mice had bilateral microrchidia (22.2%), whilst the others showed a normal testicular size. In these abnormal mouse testes, bilateral testicular tissues showed similar pathological changes.

**Figure 1:**
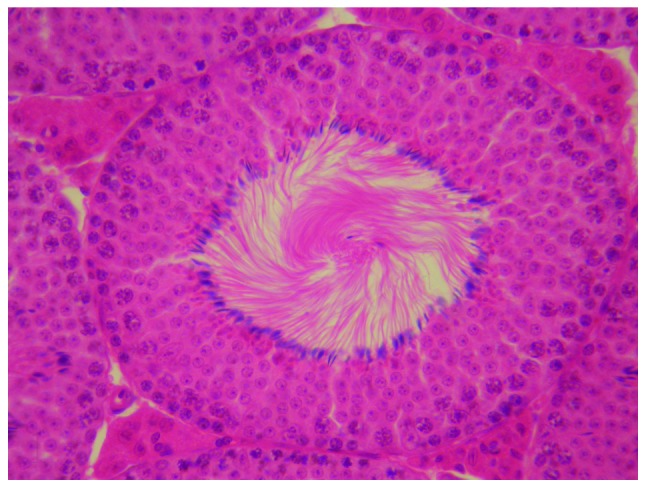
Normal spermatogenesis. The seminiferous tubule shows a clear lumen and a normal arrangement of cellular types (haematoxylin and eosin stain; magnification, ×400).

**Figure 2:**
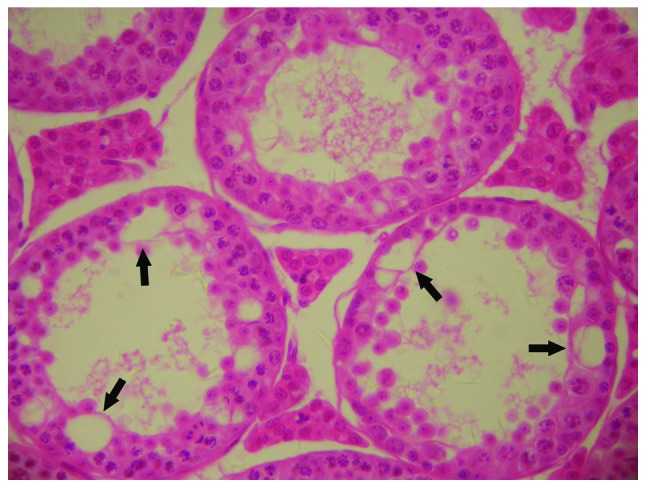
Seminiferous epithelia vacuolation (black arrows) and the absence of sperm are visible in all the tubules. Spermatogenesis was arrested at the spermatocyte stage (haematoxylin and eosin stain; magnification, ×400).

**Figure 3:**
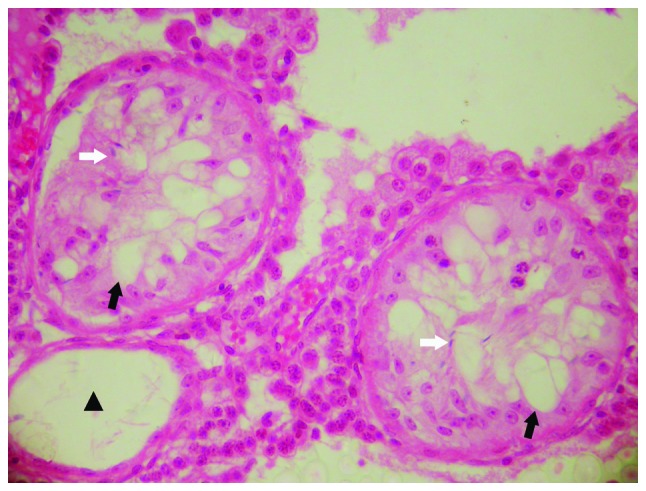
Seminiferous epithelia show severe hypospermatogenesis. Seminiferous epithelial vacuolation (black arrows) and the rare presence of sperm (white arrows) are present in tubules. ^p^The absence of seminiferous epithelium is observed in the tubule (haematoxylin and eosin stain; magnification, ×400).

**Figure 4:**
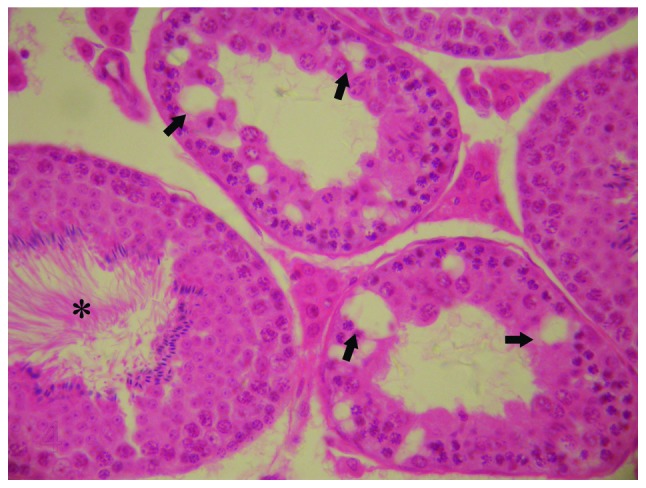
Hypospermatogenesis and seminiferous epithelial vacuolation (black arrows) are visible in the tubules. ^*^Adjacent tubule shows normal spermatogenesis (haematoxylin and eosin stain; magnification, ×400).

**Figure 5:**
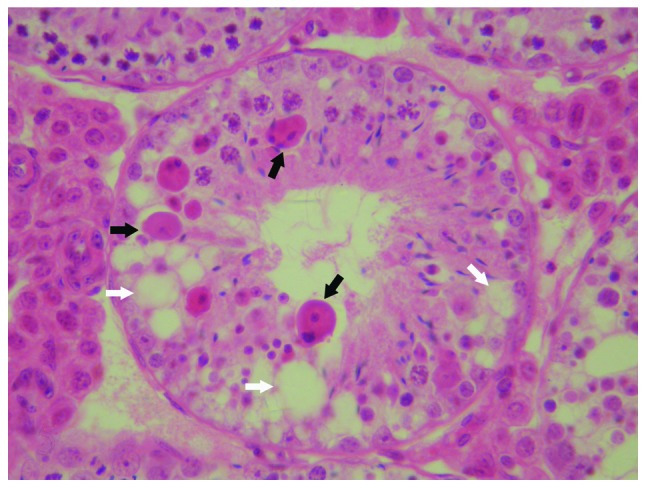
Symplasts composed of collapsed spermatids (black arrows) and seminiferous epithelial vacuolation (white arrows) are exhibited in the tubule (haematoxylin and eosin stain; magnification, ×400).

In normal testes, histological examinations demonstrated a normal arrangement of cellular components (Fig. 1). In the mice with microrchidia, testicular tissue showed that seminiferous epithelial vacuolation and the absence of sperm existed in all tubules and that spermatogenesis had arrested at the spermatocyte stage (Fig. 2). In other abnormal testes, testicular pathological changes included seminiferous epithelial vacuolation, severe hypospermatogenesis, the rare presence of sperm and the absence of seminiferous epithelium and Sertoli cells in tubules (Fig. 3). Moderate hypospermatogenisis was observed in tubules (Fig. 4), as well as seminiferous epithelial vacuolation and a small number of symplasts composed of collapsed spermatids (Fig. 5).

## Discussion

The results of the present study are, to the best of our knowledge, the first to show that normal adult male mouse testes exhibit pathological changes, with an incidence of testicular abnormality of 1.3%. The histological changes may induce spermatogenic disorders and an absence of sperm and pathological changes of the male mouse testes may cause male infertility.

Abnormalities in spermatogenesis promote germ cell apoptosis, which results in spermatogenetic arrest (8). The spermatogenetic arrest, at various stages of spermatogenesis, causes subfertility or infertility and may be associated with genetic abnormalities (9). Various aspects of spermatogenetic arrest have also been reported and correlated with various possible mechanisms of meiotic abnormalities (10). Moreover, there are a variety of factors that have been demonstrated to cause spermatogenetic arrest, including genetic mutation, environmental factors and hormone deficiency (11,12). Testis weight is also an important indicator of overall testicular health, reflecting changes in germ cell loss (13). In the present study, in which the mice were reared in the same environment and conditions, histological examination showed that spermatogenesis was arrested at the spermatocyte stage. Additionally, microrchidia may also cause spermatogenetic arrest in the adult male mouse. Autosomal recessive mutation of the microrchidia (morc) gene results in the complete arrest of spermatogenesis at an early meiotic stage (14). Hence, we hypothesized that a spontaneous mutation is a possibility but morc mutation is more likely. In addition, other possibilities require consideration, including acute febrile disease, hyperthermia and other systemic insults/diseases.

Vacuolar changes of the seminiferous epithelia were observed to occur in normal mouse testicular tissues in the present study. Similar pathological changes of testicular tissues have also been reported in previous toxicological studies (15,16). Spermatogenic epithelial vacuolation in testis has been observed to decrease the number of testicular sperm (17). Moreover, seminiferous epithelial vacuolation is common in affected tubules, particularly near the rete, indicative of a breakdown in Sertoli-germ cell junctions (18). These factors indicate that pathological changes of the seminiferous epithelia in testes may lead to hypospermatogenesis. However, the etiology of vacuolation in testicular tissue is unknown. Vacuolation may be associated with abnormal gene expression (19,20). Mouse reproductive tract diseases may also cause seminiferous epithelial vacuolation (21). Therefore, abnormal testes should be identified when using normal adult mouse testes for studies of seminiferous epithelia.

The potential limitations of the present study require consideration. Firstly, this study was a retrospective analysis. It was not possible to examine whether the pathological changes of testicular tissues correlated with changes in other reproductive organs in normal adult mice, including the epididymis, prostate, seminal vesicles and urethral anatomy. Secondly, there were no surplus samples of abnormal testicular tissues for further analysis at the molecular and genetic levels. If possible, more studies of testicular tissues are likely to not only aid the understanding of pathogenesis of abnormal testes, but also provide new information concerning the etiology of developmental defects.

In conclusion, the results show that normal adult male mice exhibit testicular pathological changes. Therefore, more attention should be paid to the possibility of abnormal testes when using normal adult male mice to establish a testicular experimental model.
